# Determination of Macro- and Microelements in the Inflorescences of Banana Tree Using ICP OES: Evaluation of the Daily Recommendations of Intake for Humans

**DOI:** 10.1155/2020/8383612

**Published:** 2020-11-16

**Authors:** Rafaela Henriques Rosa, Melina Ribeiro Fernandes, Elaine Silva de Pádua Melo, Daniela Granja Arakaki, Nayara Vieira de Lima, Luana Carolina Santos Leite, Paulo Renato Espindola, Igor Domingos de Souza, Valdir Aragão do Nascimento, Paula Fabiana Saldanha Tschinkel, Fabiane La Flor Ziegler Sanches, Valter Aragão do Nascimento

**Affiliations:** ^1^Group of Spectroscopy and Bioinformatics Applied Biodiversity and Health (GEBABS), Graduate Program on Health and Development in West Central Region, School of Medicine, Federal University of Mato Grosso do Sul, UFMS, 79070-900 Campo Grande, Brazil; ^2^Institute of Physics of the Federal University of Mato Grosso do Sul, Campo Grande, Mato Grosso do Sul, Brazil; ^3^Faculty of Pharmaceutical Sciences, Food and Nutrition, Federal University of Mato Grosso do Sul, 79070-900 Campo Grande, Brazil

## Abstract

The inflorescence of *Musa paradisiaca*, known as “banana heart” is a structure that includes flowers and bracts of banana, commonly used as food source worldwide. The aims of this study were (1) to determine the mineral components of *Musa paradisiaca* and (2) to compare the obtained results with previously reported data of Recommendation Dietary Allowances (RDAs) and edible plant permissible limits set by FAO/WHO. The samples were digested using microwave-assisted equipment, while elemental contents were determined by inductively coupled plasma optical emission spectroscopy (ICP OES). Metal (Mg, Ca, Cr, Ni, Cu, Fe, and Zn) and nonmetal (S and P) contents were detected. According to RDA, the inflorescences could be excellent sources of Mg, P, Cr, Cu, Zn, and Fe for females, males, and pregnant women, all age 31–50 y, as well as children (4–8 y). Bracts are good source of Zn for male and pregnant women and good source of Fe for children. All the samples contained considerable amounts of Mg, Ca, P, Ni, Cu, Zn, and Fe, which were quite low to induce deleterious effects (UL). FAO/WHO limits for edible plants have not yet been established for S, P, Mg, and Ca, but Ni and Zn are below of those limit values. However, Cr and Cu concentrations are higher than the values established for edible plants and may pose a threat to human health. Farmers should be encouraged by government agencies, not only for sustainability of production but also to ensure the storage and trade of banana tree inflorescence.

## 1. Introduction

A banana is an edible fruit produced by the banana tree. There are considerable differences concerning cultural practices and utilizations of the banana fruit and its peel by populations [1]. The fruits grow in clusters hanging from the top of the plant. *Musa paradisiaca* (plantain) is the accepted name for the hybrid between *Musa acuminata* and *Musa balbisiana* [2]. *M. paradisiaca* is a herbaceous plant of the genus *Musa* being sourced from tropical and subtropical countries such as Sri Lanka and eastern India and regions of other countries as China and Australia [3]. There is a great diversity of cultivated bananas. Thus, many other names are synonyms of *M. paradisiaca*, such as *M. corniculata* Lour [3].

The fruit of *M. paradisiaca* is a good source of potassium, calcium, and phosphorus and is also a rich source of iron and vitamins C and E [4, 5]. *M. paradisiaca* has potential health benefits [6] as well as cancer chemoprevention activities [7]. Several parts of the banana tree serve as raw material for making ropes or are used in religious ceremonies and clothing manufacturing [8]. According to the Food and Agriculture Organization of the United Nations (FAO) estimates, India produced 29 million tons of banana per year on average between 2010 and 2017. China follows it with 11 million tons, the Philippines with an annual average of 7.5 million tons from 2010 to 2017, and Brazil an average of 7 million tons [9].

In Brazil, due to socioeconomic conditions and search for healthier nonconventional foods, banana inflorescences are used as food by the population; farmers even discard this part during the collection of fruits [10]. The inflorescence is constituted of the mating and floral axis (raque), where the flowers are inserted in pence consisting of two horizontal and parallel rows. The banana tree's inflorescence is popularly known as the “banana heart” or “navels,” which is used to make salads or used in the form of dry flour as food in several Brazil regions. The inflorescences of the banana tree (male flowers and bracts) are commonly used as vegetables for cooking in Laos, Thailand, China, Burma, Philippines, Sri Lanka, Vietnam, and India cooking methods vegetables were adopted [11].

Due to the indiscriminate use of fertilizers and pesticides for crop protection and pest control, fruits [12] and the banana tree are being contaminated by heavy metals [13]. High levels of Na, Pb, Cr, Fe, Mn, Cd, Zn, and Se in the banana may have been attributed to the repeated use and addition of residues as fertilizers in agricultural areas of banana [14]. Besides, studies on banana plants that are grown in industrial areas have elevated levels of Cd, Co, Cu, Fe, Pb, Ni, and Zn [15]. According to Romero-Estévez et al. [16], there is no significant health risk to the consumer associated with banana fruits with contamination levels of Cd and Ni, but there is Pb risk for toddlers. In fact, the heavy metal concentration in fruits, legumes, vegetables, and animals is influenced by the environmental factors (agriculture fertilizers, pollution, climate, and physicochemical properties and soil type) where they are growing [17–20]. Toxic elements (Cd, Pb, Cu, and Zn) are present in herbs [21], animals, and other types of food according to proven studies in several countries [22–24]. Therefore, it is essential to monitor food quality once plants accumulate heavy metals [25]. Macro- (Ca, P, Na, S, K, and Mg) and microelements (B, Cl, Cr, Cu, F, I, Fe, Li, Co, Mn, Mo, Ni, Se, Na, V, and Zn) play decisive roles in the human metabolism and are necessary for good health [26, 27]. However, large amounts of these nutrients through oral ingestion can cause health risks to farmers and consumers.

Each element's potential contribution to healthy people was stipulated by dietary reference intakes (DRIs) [28]. DRIs encompass four types of nutrient reference values, including the RDA, which is an average daily dietary intake level; the Adequate Intake (AI) being a value based on experimentally derived intake levels or approximations of observed mean nutrient intakes by a group (or groups) of healthy people; the Tolerable Upper Intake Level (UL), meaning the highest level of a daily nutrient that is likely to pose no risk or adverse effects to almost all individuals in the general population; and the Estimated Average Requirement (EAR) is the intake level for a nutrient at which the need of 50 percent of the people will be met).

The quantification of metals and nonmetals in human foods can be performed using analytical methods. Thus, the value of quantification in samples by ICP OES can be used to stipulate the potential contribution of each element to healthy individuals established by the DRIs [29–32]. In fact, from spectroscopic techniques and stipulated values of daily intake (RDA/AI and UL), it is possible to perform calculations that estimate the necessary intake of each element required to maintain human health or may cause toxicity [33, 34]. In some countries, the environmental factors in unconventional food plants are not subject to strict control. Food security can be provided by looking at the information on molecular composition, process intermediates, and quality parameters, which can be assessed by infrared spectroscopy in both liquid and gas phases [35]. In addition, molecular identification in biological sample in gas phase using infrared spectroscopy can assist in diagnosing diseases in the human body or to ensure its safety [36].

Motivated by the manuscript published by Oyeyinka and Afolayan [33], which emphasizes that the peel and its derivative extract, as well as the flesh of *M. sinensis* L. and *M. paradisiaca* L., are relevant to human nutritional, we believe the inflorescence and flowers of *M. paradisiaca* are also essential for health. There is no information on the concentration of metals and nonmetals in the inflorescence of other banana species such as *M. paradisiaca* L., which is consumed by the indigenous, urban, and rural population of central-western Brazil and other countries [11]. This study aimed to determine and compare for the first time the contents of the metals (Mg, Ca, Cr, Ni, Cu, Fe, and Zn) and nonmetals (S and P) in the inflorescence of *M. paradisiacal* L. (male flowers and bracts) with the specification limits of RDA, AI, and UL values for male, female, children, and pregnancy [28]. In addition, comparisons were also made, considering the limits established for edible plant permissible limits set by FAO/WHO [37]. It is very important to know the concentration of heavy metals and nonmetals in plants and fruits to estimate their role as sources of components in the diet and workers' and producers' safety. The collection of the samples was performed several times from in the urban area of the city of Campo Grande, Brazil. The samples of the inflorescence of *M. paradisiacal* L. selected for our research underwent a microwave-assisted digestion process, after which ICP OES determined the macrocontent and microelements.

## 2. Materials and Methods

### 2.1. Collection and Identification of Vegetable Material

**Figure 1:**
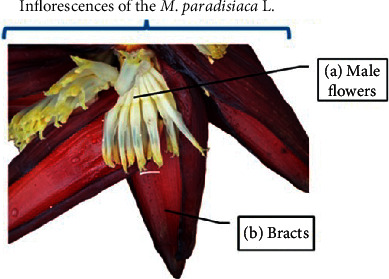
Inflorescence of *M. paradisiaca* L. (a) Male flowers and (b) bracts.

For the analysis of chemical elements, a total of 16 samples of inflorescences of banana trees (*M. paradisiaca L*., Musaceae) were collected in May 2019 in a smallholding near the urban area of the city of Campo Grande, Mato Grosso do Sul, Brazil. Samples of this banana tree species have already been identified by the herbarium of the Federal University of Mato Grosso do Sul on 26/06/2016, deposit No. 53972 CGMS. Male flowers are produced within bracts near the apex of the floral stalk (Figure 1). In accordance with Brazilian law, this research was registered in the National Genetic Resource Management System and Associated Traditional Knowledge (SisGen, No. A7716EC).

### 2.2. Sample Preparation and Digestion

A quantity of 100 g of flowers (bracts) of several banana trees (about 20) was collected and mixed for analysis. The flowers and bracts were set to dry in an oven at 50°C for 72 hours until their weights stabilized. The dried samples of flowers and bracts were crushed separately with a portable stainless steel electric grinder to obtain a very fine powder. About 0.250 g of flowers (as well as 0.250 g bracts) were weighed on an analytical balance, transferred inside Teflon vessels (in triplicate), and 3 ml of nitric acid (HNO_3_ 65% Merck), 2 ml of hydrogen peroxide (35% H_2_O_2_, Merck), and 1 ml of ultrapure water (conductivity 18.2 MΩcm produced Millipore, Water Purification System Milli-Q Biocel, Germany) were added. For the calibration blank, 1 ml of ultrapure water and 2 ml of nitric acid in 1 ml of H_2_O_2_ was used.

Once you have all the steps mentioned above in place, the mixture was allowed to remain in the open air for 10 min predigestion and then digested using a microwave digestion equipment (BERGHOF Products + Instruments GmbH—Speedwave 4—Microwave Digestion System). All digestion processes were carried out using the operating program specified in Table 1.

### 2.3. Elemental Analysis by ICP OES Technique

After the microwave system's digestion procedure, the contents of the vessels were transferred to the 50 ml Falcons vessel and then filled to 30 ml with ultrapure water. Also, the elements were determined by the technique of ICP OES (Thermo Fisher Scientific, Bremen, Germany, iCAP 6300 Duo). The selected emission lines (wavelength in nm) for determining elements in flowers, bracts, and operating conditions of ICP OES are summarized in Table 2.

### 2.4. Calibration Curves

For the ICP-OES method, multielement stock solutions containing 1000 mg/L of Al, As, Ca, Cd, Co, Cr, Cu, Fe, Mg, Mn, Mo, Na, Ni, P, S, V, Se, and Zn were obtained from SpecSol (SpecSol, Quimlab, Brazil), and analytical calibration standards were prepared. For each element detected (Table 3), a limit of quantification (LOQ), limit of detection (LOD), and correlation coefficient (*R*^2^) were established according to [38]. A blank and a seven-point calibration curve were generated using the following concentrations: 0.01, 0.02, 0.05, 0.2, 1.0, 2.0, and 5.0 mg/L of the all element standard. Triplicate analyses were performed for each sample.

The accuracy was also verified by addition and recovery, where it was found that our quantification analyses for each element were detected with good precision (i.e., standard deviation (SD) between 0.01 and 0.3), as explained in Table 4.

### 2.5. Statistical Analysis

The statistical package for social sciences (SPSS), version 18.0 (SPSS Inc. Chicago, IL, USA), was used to study the differences in contents between flowers and bracts of the banana tree's inflorescence. Results are presented as mean ± standard deviation. The Kolmogorov-Smirnov test was used to verify the normality of the data of the contents of flowers and bracts obtained by ICP OES. After checking for normality, the Student's *t* test was used to compare the means. It was adopted *p* < 0.05 as a level of significance.

## 3. Results and Discussion

Results showed that seven metal (Mg, Ca, Cr, Ni, Cu, Fe, and Zn) and two nonmetal (S and P) contents were detected in the inflorescence of the banana tree (Table 5). Each sample was analyzed three times by the ICP OES, and the results were expressed as mean ± SD. The elements as sodium (Na), selenium (Se), vanadium (V), manganese (Mn), molybdenum (Mo), cobalt (Co), cadmium (Cd), aluminium (Al), and arsenium (As) are below the limit of detection (LOD). According to results of Student's *t* test (Table 5), there was a significant difference between the contents of flowers and bracts for sulfur (S), phosphorus (P), calcium (Ca), copper (Cu), and zinc (Zn) (*p* < 0.001). On the other hand, there was no statistically significant difference between magnesium (Mg), chromium (Cr), nickel (Ni), and iron (Fe) elements in leaves and bracts (*p* > 0.05) between flowers and bracts.

Although some elements are below the detection limit, some have an accumulative effect or lead to health problems in humans due to the low or high concentrations [39].  Table 6 list the levels of nonmetals and metals quantified (mg/100 g) in the inflorescence of the banana tree compared to the limit specification of RDAs, AI, and UL values of males (31–50 y) and females (31–50 y), children (4–8 y), and pregnant women (31–40 y) [28]. Besides, all values obtained in this manuscript were compared with the permissible limits set by the FAO/WHO for edible plants [36].

This manuscript adopted the Nutrition Content Claims based on FDA, which proposed that foods with 10–19% of the daily value per portion are “good source of” nutrition, while foods that contain 20% or more than daily values per portion are considered “excellent source of” nutrition [40]. As shown in Table 6, the concentration of nonmetal in the flowers and bracts decrease in the order P > S and metal Ca > Mg > Zn > Fe > Cu > Ni > Cr. The discussions on the concentration of each element obtained in the flowers and bracts in this manuscript are present[[parms resize(1), pos(50,50), size(200,200), bgcol(156)]]ted in flowers and bracts of the banana tree's inflorescence were 90.100 ± 0.859 mg/100 g and 75.597 ± 0.290 mg/100 g (Table 6). Sulfur does not have an established RDA [28]. Although there is no known dietary requirement for Sulfur, some experts recommend 800–900 mg/day of Sulfur for adults, 1,200 mg/day for pregnant women, and 1,500 mg/day for patients with osteoarthritis [41].

The safe limit for Sulfur has not been established by UL for males, females, children, and pregnant women (Table 6). The chemical element sulfur is not toxic; however, some sulfur derivates are, such as sulfur dioxide and hydrogen sulfide. In humans, sulfate intake exceeds approximately 3 g/day due to sulfate ingestion in food and water [42]. On the other hand, the sulfur content of food can be estimated using the amino acids as methionine and cysteine [43].

FAO/WHO limits for edible plants have not yet been established for S [37]. In fact, studies on the intake of sulfur-containing foods are scarce. There are no recognized toxic effects of dietary sulfur. It is not possible to affirm that banana inflorescence does not represent a risk of adverse health effects of males, females, children, and pregnant women.

According to data in Table 6, the contents of phosphorous detected in the flowers were 307.389 ± 3.601 mg/100 g, which corresponds to 43.912 ± 0.514% of the total intake of 700 mg/day of RDA for male, female, and pregnant women and 61.477 ± 0.72% for children. Besides, the concentration of P detected in bracts was 282.398 ± 0.551 mg/100 g, corresponding to 40.34 ± 0.071% of the RDA for males, females, and pregnant women and 56.479 ± 0.110% for children. Thus, flowers and bracts can be considered as excellent sources of P (more than 20% of DRI) for adults, pregnant women, and children aged 4–8 years (Table 6).

The level of P determined in the flowers and bracts of the inflorescence does not represent a health risk because it is below the UL for P's consumption in men, women, children, adolescents, and pregnant women (3,000–4,000 mg/100 g) (Table 6). For edible plants, the FAO/WHO limits have not yet been established for P [36]. Foods rich in phosphorus are beneficial to human health. However, excess phosphorus can cause body changes and have critical negative effects on bone health [44].

In Table 6, the concentration of magnesium detected in flowers was 171.602 ± 2.261 mg/100 g, which corresponds to 40.71 ± 0.53% of RDA for males (420 mg/day), 53.43 ± 0.70% of RDA for females (320 mg/day), 131.53 ± 1.73% of RDA for children (110 mg/day), and 47.50 ± 0.62% of RDA for pregnant women (360 mg/day). The concentration of Mg in bracts detected (172.686 ± 2.028 mg/100 g) correspond to 40.95 ± 0.48% of the RDA for male, 53.75 ± 0.63% of RDA for females, 132.0 ± 1.56% of the RDA for children, and 47.77 ± 0.56% of RDA for pregnant women. Therefore, the flowers and bracts are an excellent Mg source for people in this age group of 4 to 50 years.

The concentration of Mg was found to be the same in flowers and bracts, which is below the permissible level of 350 mg/day set by UL [28]. Therefore, the inflorescence of the banana tree does not represent a risk of adverse health effects for males, females, children, adolescents, and pregnant women. There is no limit for Mg in edible plants established by FAO/WHO.

Studies have demonstrated the effect of Mg supplementation in the treatment of some diseases [45]. However, Mg toxicity occurs during intravenous Mg treatment [46]. To date, we have not found published papers on magnesium toxicity due to food intake. In clinical practice, the magnesium diet and supplementation appear to be reliable [47]. Studies on supplementing 360 mg/day of Mg in pregnancy demonstrated efficacy concerning ameliorating muscle cramps [48].

The level of calcium in the flowers was 285.444 ± 5.412 mg/100 g (Table 6), corresponding to 28.544 ± 0.542% of the 1000 mg/day recommended for men by AI for male, females, and pregnant women and 35.680 ± 0.676% of the AI for children (800 mg/day). Bracts (380.632 ± 4.066 mg/100 g) correspond to 38.063 ± 0.406% of the AI for males, females, and pregnant women and 47.579 ± 0.508% for children. Consequently, it is concluded that the flowers and bracts are excellent Ca source for people in the age group of 31 to 50 years and children. In recent years, based on various clinical practice guidelines, calcium has been supplemented taken for men and women, children, and adults to improve their skeletal health [49].

The Ca level in Table 6 (flowers and bracts) was below the UL values for Ca's consumption in males and females (2,500 mg/day), with no risk of adverse health effects. For edible plants, the FAO/WHO limits have not yet been established for Ca [37]. According to Bernett et al. [50], calcium intake's toxic effects have only been reported when the calcium is given the carbonate in very high doses.

According to data in Table 6, the chromium concentration found in flowers (0.027 ± 0,006 mg/100 g) corresponds to 77.142 ± 17.14% of the AI for male (0.035 mg/day), 108.0 ± 10.8% of the AI for females (0.025 mg/day), 180.0 ± 40% of AI for children (0.015 mg/day), and 90 ± 20% for pregnant women (0.030 mg/day). The content found in bracts (0.047 ± 0.009 mg/100 g) corresponds to 134.285 ± 25.714% of the AI for male, 188.0 ± 36.0% of the AI for females, 313.33 ± 60% for children, and 156.66 ± 30% for pregnant women. After comparison of the concentration of Cr in flowers and bracts with those values proposed by the AI, it is concluded that the flowers and bracts are an excellent source of Cr for people in the age group of 31 to 50 years and children aged 4–8 years.

There are no established limits by UL for Cr for these populations (Table 6). However, it was found that the concentration of Cr in flowers and bracts was higher than the allowed limit established by FAO in edible plants (0.002 mg/100 g) [37]. The National Academy of Sciences has determined a safe and adequate daily intake for Cr (III) in adults of 50–200 micrograms per day, respectively [51]. Therefore, special care should be taken with respect to the ingestion of this vegetable in large quantities.

The nickel concentration obtained in the flowers and bracts of the Banana tree's inflorescence was 0.072 ± 0.003 mg/100 g and 0.070 ± 0.004 mg/100 g (Table 6). There is no limit for Ni concentration established by RDA and AI values for people. However, the concentration of Ni in flowers and bracts are below UL values for adults aged 31–50 years (1.0 mg/day) and children aged 4–8 years (0.3 mg/day).

The permissible limit set by FAO/WHO for edible plants was 0.163 mg/100 g (Table 6) [36]. According to the DEPARTMENT of HEALTH AND HUMAN SERVICES, Public Health Service Agency for Toxic Substances and Disease Registry, food is the major source of nickel exposure for adults and children (dietary intake, 0.170 mg/day) [52]. Studies on Nickel's dietary intake assessment indicate that the diet provides less than 0.2 mg/day [53]. Thus, it is found that the inflorescence of the banana tree does not represent a risk of adverse health, since the accumulated Ni in flowers and bracts are below the limits set [28, 52, 53].

The content detected of copper in flowers (0.385 ± 0.007 mg/100 g) correspond to 42.77 ± 0.77% of the RDA for male and females (0.9 mg/day), 87.50 ± 1.59% for children (0.440 mg/day), and 38.50 ± 0.70% for pregnant women (1 mg/day). On the other hand, the content detected of Cu in bracts was 0.318 ± 0.008 mg/100 g, which correspond to 35.33 ± 8.88% of the RDA for male and females, 72.272 ± 1.81% for children, and 31.80 ± 0.80% for pregnant women. According to these results, flowers and bracts are an excellent source of Cu for people in the age group of 31 to 50 years and children aged 4–8 years.

Cu's concentration in the banana inflorescence is lower than the values established by UL for adults, children, and pregnant women (3–10 mg/day). However, it is higher than the concentration of edible plants established by FAO/WHO (0.3 mg/100 g) [37]. High concentration copper in foods can cause adverse long-term effects in humans [54]. Exposure to excessive copper levels can result in kidney damage, anemia, immunotoxicity, and developmental toxicity [55].

The content of Zinc in flowers was 2.565 ± 0.037 mg/100 g (Table 6), whose percentage was 24.14 ± 0.336% of value established by RDA for male and pregnant women (11 mg/day), 32.06 ± 0.46% for females (8 mg/day), and 51.30 ± 0.74% for children (5 mg/day). Zn concentration in bracts (1.807 ± 0.014 mg/day) corresponded to 16.427 ± 0.127% of value established by RDA for male and pregnant women, 22.587 ± 0175% for females, and 36.14 ± 0.28% for children. Flowers are an excellent source of Zn to males, females, children, and pregnant women. The bracts are a good source of Zn for males and pregnant women; they are excellent Zn sources to females and children.

The Zn level in Table 6 is below the UL for the consumption of Zn in males, females, and pregnant women (40 mg/day) and children (12 mg/day). The permissible limit set by FAO/WHO for Cu in edible plants was 2.74 mg/100 g [37]. Thus, the plant accumulated Zn below this limit.

Dietary zinc levels are recommended during pregnancy and lactation [56]. Besides, supplementation with zinc showed efficacy in the treatment of some diseases [57].

In Table 6 the concentration of iron in flowers (1.84 ± 0.520 mg/100 g) correspond to 23.05 ± 6.50% of the RDA for male (8 mg/day), 10.24 ± 0.28% of RDA for females (18 mg/day), 18.40 ± 5.2% for children (10 mg/day), and 6.81 ± 1.92% for pregnant women (27 mg/day). The concentration of Fe for bracts (1.66 ± 0.440 mg/100 g) corresponds to 20.68 ± 5.5% of the RDA for male, 9.22 ± 2.44% of RDA for females, 16.60 ± 4.4% of the RDA for children, and 6.14 ± 1.62% of RDA for pregnant women. After comparison of the concentration of Fe in flowers and bracts with those values proposed by the RDA [28], it was found that the flowers and bracts are an excellent source of Fe for males. On the other hand, flowers are a good source of Fe for females and children. Bracts are a good source of Fe for children, respectively.

Fe concentration in flowers and bracts is below the permissible level of 45 mg/day set by UL [28]. Therefore, the banana tree's inflorescence does not represent a risk of adverse health effects for males, females, children, and pregnant women. In fact, the ingestion of less than 20 mg/kg of elemental iron is nontoxic; however, ingestion of more than 60 mg/kg can result in severe toxicity and lead to severe morbidity and mortality [58]. Fe's concentration in the banana bracts and flowers is close to the value allowed by FAO/WHO for edible plants, which is 2.0 mg/100 g [37].

In the paper published by Oyeyinka and Afolayan [33], elements as Ca, Mn, P, Na, Zn, Mg, Cu, Fe, and K were quantified in the peel, flesh, and peel extract of *M. sinensis* and *M. paradisiaca*. On the other hand, in our paper, the inflorescence and flowers of the *M. paradisiaca* were quantified P, S, Ca, Mg, Zn, Fe, Cu, Ni, and Cr. Thus, inflorescence and banana flowers can be considered as food for people.

## 4. Conclusions

New information on the concentration of macro- and microelements in the inflorescence of *Musa paradisiaca* was obtained and compared with recommended values (RDA/AI), tolerable upper intake levels (ULs), and edible plants. Flowers and bracts are an excellent source of Mg, Ca, P, Cr, Cu, and Zn for males and females.

For elements such as sulfur and nickel, the present study showed that there are gaps regarding knowledge of the levels in which nutrients can be ingested (RDA/AI), especially children and pregnant women.

There are no limits established to S, Mg, Ca, and P by FAO/WHO for edible plants. However, the concentrations of S were low relative to values stipulated by experts.

UL has not yet established the limits for chromium, sulfur, and nickel. However, the present study showed that *M. paradisiaca*'*s* inflorescence accumulates chromium and copper above the limit set by FAO/WHO (edible plant). On the other hand, the concentration of Mg, Ca, P, Ni, Cu, Zn, and Fe was low relative to the UL's values. The concentration of Ni and Zn is below the amount established by FAO/WHO in edible plants.

The data obtained would serve as a tool for deciding the dosage of this vegetable with nutritional purposes. However, in some cases, they carry very high content of toxic metals whose main reason is environmental factors. Therefore, special care should be taken regarding the daily intake of this unconventional food. Prolonged ingestion of metals such as chromium, copper, nickel, and zinc can cause deleterious health effects in humans.

The knowledge of the nonconventional plants has an economic interest and involves a global health problem. This paper's results and discussion would represent further valid contributions to the scientific research, farmers, and other stakeholders as governmental and nongovernmental organizations (NGOs) and society at large. Research on the inflorescence of other banana species can be conducted by opening doors for new food products.

## Figures and Tables

**Table 1:** Microwave digestion parameters.

Step	Power (W)	Temperature (°C)	Ramp time (min)	Hold time (min)	Pressure (Bar)
1	1305	170	5	10	35
2	1305	200	1	15	35
3	0	50	1	10	0
4	0	50	1	10	0
5	0	50	1	1	0

**Table 2:** Instrumental analytical conditions for ICP OES of element analyses.

Parameters	Setting
RF power (W)	1250
Sample flow rate (L min^−1^)	0.45
Plasma gas flow rate (L min^−1^)	12
Integration time (s)	5
Stabilization time (s)	20
Pressure of nebulization (p si)	20
Plasm view	Axial
Gas (99.999%)	Ar
Analytical wavelength (nm)	Al 308.215 nm, As 193.759 nm, Ca 422.673 nm, Cd 228.802 nm, Co 228.616 nm, Cr 267.716 nm, Cu 324.754 nm, Mg 279.553 nm, Fe 259.940 nm, K 766.490 nm, Mn 257.610 nm, Mo 202.030 nm, Na 589.592 nm, Ni 231.604 nm, P 177.495 nm, S 180.731 nm, Se 196.090 nm, V 309.311 nm, Zn 213.856 nm

**Table 3:** Parameters of calibration obtained external calibration: correlation coefficient (*R*^2^), LOD, and LOQ by using ICP OES.

Elements	^*∗*^ *R* ^2^	^*∗∗*^LOD (mgL^−1^)	^*∗∗∗*^LOQ (mgL^−1^)
Al	0.9996	0.001	0.003
As	0.9999	0.001	0.004
Ca	0.9999	0.00009	0.0001
Cd	0.9998	0.00008	0.0003
Co	0.9999	0.002	0.0008
Cr	0.9999	0.002	0.006
Cu	0.9997	0.002	0.006
Fe	0.9999	0.0005	0.002
K	0.9996	0.0001	0.0003
Mg	0.9999	0.00002	0.00006
Mn	0.9999	0.0001	0.003
Mo	0.9999	0.0003	0.001
Na	0.9997	0.0001	0.0005
Ni	0.9999	0.0005	0.002
P	0.9999	0.003	0.01
S	0.9999	0.002	0.006
Se	0.9994	0.0009	0.003
V	0.9999	0.0003	0.0009
Zn	0.9996	0.00009	0.003

^*∗*^ *R* ^2^: correlation coefficient; ^*∗∗*^LOD: limit of detection; ^*∗∗∗*^LOQ: limit of quantification.

**Table 4:** Addition and recovery tests (*n* = 3).

Analyte	Added (mg/L)	Obtained value (mg/L)	Recovery (%)
Al	1.00	1.00 ± 0.05	100.00
As	1.00	0.89 ± 0.01	89.00
Ca	1.00	1.07 ± 0.06	107.00
Cd	1.00	0.98 ± 0.12	98.00
Co	1.00	1.03 ± 0.10	103.00
Cr	1.00	1.06 ± 0.30	106.00
Cu	1.00	1.05 ± 0.20	105.00
Fe	1.00	0.99 ± 0.10	99.00
K	1.00	0.94 ± 0.20	93.00
Mg	1.00	1.09 ± 0.20	109.00
Mn	1.00	1.03 ± 0.10	103.00
Mo	1.00	0.92 ± 0.03	92.00
Na	1.00	0.90 ± 0.20	90.00
Ni	1.00	0.90 ± 0.04	90.00
P	1.00	0.93 ± 0.05	93.00
S	1.00	0.90 ± 0.03	90.00
Se	1.00	1.03 ± 0.06	103.00
V	1.00	0.92 ± 0.02	92.00
Zn	1.00	1.08 ± 0.05	108.00

The results are expressed as mean ± SD.

**Table 5:** Mineral composition of the flowers and bracts in the banana inflorescence by ICP OES (mg/100 g).

Available elements	Flowers (mg/100 g)	Bracts (mg/100 g)	*p* value
S	90.100 ± 0.859	75.597 ± 0.290	0.0001^*∗*^
P	307.389 ± 3.601	282.398 ± 0.551	0.001^*∗*^
Mg	171.602 ± 2.261	172.686 ± 2.028	0.570
Ca	285.444 ± 5.412	380.632 ± 4.066	0.0001^*∗*^
Cr	0.027 ± 0.016	0.047 ± 0.009	0.140
Ni	0.072 ± 0.003	0.070 ± 0.004	0.998
Cu	0.385 ± 0.007	0.318 ± 0.008	0.0001^*∗*^
Zn	2.565 ± 0.037	1.807 ± 0.014	0.0001^*∗*^
Fe	1.844 ± 0.052	1.655 ± 0.044	0.008

Values are mean ± SD. ^*∗*^Values along column are significantly different (*p* < 0.05).

**Table 6:** Nonmetals and heavy metals quantified in banana inflorescences (flowers and bracts) compared to nutritional recommendations for adults, children, and pregnancy.

	Flowers (mg/100 g)	Bracts (mg/100 g)	Males 31–50 y	females 31–50 y	Males/females 31–50 y	Children 4–8 y		Pregnancy 31–50 y		Edible plant
			1RDA/AI^*∗*^ (mg/day)	1RDA/AI^*∗*^ (mg/day)	UL (mg/day	1RDA/AI^*∗*^ (mg/day)	UL (mg/day)	1RDA/AI^*∗*^ (mg/day)	UL (mg/day)	2FAO/WHO (mg/100 g)
*Nonmetals*										
S	90.100 ± 0.859	75.597 ± 0.290	ND	ND	ND	ND	ND	ND	ND	ND
P	307.389 ± 3.601	282.398 ± 0.551	700	700	4,000	500	3,000	700	3,500	ND

*Metals*										
Mg	171.602 ± 2.261	172.686 ± 2.028	420	320	350	130	110^*∗∗*^	360	350^*∗∗*^	ND
Ca	285.444 ± 5.412	380.632 ± 4.066	1,000^*∗*^	1,000^*∗*^	2,500	800^*∗*^	2,500	1,000^*∗*^	2,500	ND
Cr	0.027 ± 0.006	0.047 ± 0.009	0.035^*∗*^	0.025^*∗*^	ND	0.015^*∗*^	ND	0.030^*∗*^	ND	0.002
Ni	0.072 ± 0.003	0.070 ± 0.004	ND	ND	1	ND	0.3	ND	1	0.163
Cu	0.385 ± 0.007	0.318 ± 0.008	0.9	0.9	10	0.440	3	1	10	0.3
Zn	2.565 ± 0.037	1.807 ± 0.014	11	8	40	5	12	11	40	2.74
Fe	1.84 ± 0.520	1.66 ± 0.440	8	18	45	10	40	27	45	2.0

*Note.* ND = not determined; ^1^Recommended Dietary Allowances (RDA) [28]. ^*∗*^The value for AI is used when there are no calculated values for the RDA. ^2^FAO/WHO (1984) (mg/100 g) [37]. ^*∗∗*^ The ULs for magnesium represents intake from a pharmacological agent only and do not include intake from food and water.

## Data Availability

The data used to support the findings of this study are included and referred within the article.
